# Tuneable carbon dots coated iron oxide nanoparticles as superior *T*_1_ contrast agent for multimodal imaging

**DOI:** 10.5599/admet.2790

**Published:** 2025-06-18

**Authors:** Anbazhagan Thirumalai, Palani Sharmiladevi, Koyeli Girigoswami, Alex Daniel Prabhu, Agnishwar Girigoswami

**Affiliations:** 1 Medical Bionanotechnology, Faculty of Allied Health Sciences (FAHS), Chettinad Hospital & Research Institute (CHRI), Chettinad Academy of Research and Education (CARE), Kelambakkam, Chennai, TN-603103, India; 2 Medical Bionanotechnology Lab, Department of Obstetrics and Gynaecology, Centre for Global Health Research, Saveetha Medical College, Saveetha Institute of Medical and Technical Sciences, Thandalam, Chennai, 602105, India; 3 Department of Radiology, Chettinad Hospital & Research Institute (CHRI), Chettinad Academy of Research and Education (CARE), Kelambakkam, Chennai, TN-603103, India

**Keywords:** Magnetic nanoparticles, super paramagnetic iron oxide nanoparticles, hydrothermal method, *T*_1_ weighted magnetic resonance imaging, fluorescence imaging, diagnosis

## Abstract

**Background and purpose:**

Multifunctional hybrid nanoparticles garner heightened interest for prospective biomedical applications, including medical imaging and medication administration, owing to their synergistic benefits of constituent components. Therefore, we demonstrated an optimized protocol for synthesizing magnetofluorescent nanohybrids comprising fluorescent carbon dots with magnetic nanoparticles.

**Experimental approach:**

Carbon dot-coated iron oxide nanoparticles (CDs@Fe_2_O_3_) were synthesized with varying citric acid concentrations by a one-pot hydrothermal synthesis method for the development of a low-cost and biocompatible contrast agent (CA) for enhanced multimodal imaging (fluorescent and *T*_1_ and *T*_2_ weighted magnetic resonance imaging (MRI)) to replace the conventional CAs.

**Key results:**

The physicochemical characterization of the synthesized CDs@Fe_2_O_3_ revealed that 3 g of citric acid used for the synthesis of nanoparticles, keeping Fe(II) and Fe(III) ratio 1:2 provides higher stability of -78 mV zeta potential, saturation magnetization of 24 emu/g, with a hydrodynamic diameter of 68 nm. Carbon coating affects surface spins on Fe_2_O_3_, resulting in fewer surface-based relaxation centres, making *T*_1_ relaxation relatively more prominent. Furthermore, the surface-engineered iron oxide NPs are efficient in producing both *T*_1_ and *T*_2_ weighted MRI as well as fluorescence-based imaging. The molar relaxivity and molar radiant efficiency derived from phantom studies demonstrate their effectiveness in multimodal medical imaging. The cytotoxicity assay, haemolysis assay, haematology, and histopathology studies show that the optimized CDs@Fe_2_O_3_ are biocompatible, haemocompatible, and negligibly toxic.

**Conclusion:**

We can conclude the significant potency of CDs@Fe_2_O_3_ for multimodal diagnosis.

## Introduction

Nanoparticles, with their unique physical and chemical traits, offer a promising way to overcome the limitations of traditional therapies for life-threatening diseases like cancer [1,2]. Their ability to be designed for targeted therapy is a key advantage, as it allows for more precise delivery of therapeutics to the diseased sites. This precision is crucial in cancer therapy, as it can minimize the toxic effects on healthy tissues [3,4]. Systems based on inorganic metal nanoparticles offer a wide range of surface modifications, which can be utilized for imaging and targeting through molecular interactions, thereby enhancing their theranostic potential [5-7]. Magnetic nanoparticles (MNPs) are a fascinating type of nanomaterial that has been broadly researched for numerous technological applications [8-10]. In biomedical fields, MNPs are employed to produce heat for hyperthermia treatments and facilitate the remote management of targeted payload delivery, including serving as magnetic resonance imaging (MRI) contrast agents (CAs) [11-14]. Among magnetic materials, iron oxide NPs have received significant interest in the field of nanomedicine, which are obtained in three natural forms such as α-Fe_2_O_3_ (hematite), γ-Fe_2_O_3_ (maghemite), and Fe_3_O_4_ (magnetite) [15,16]. These various kinds of iron oxide NPs demonstrate several encouraging characteristics, such as biocompatibility, minimal toxicity, decreased susceptibility to oxidation, enhanced stability in magnetic behaviour, the potential to transfer superparamagnetic state through reduced particle size, easy synthesis, and surface modifications. Superparamagnetic NPs offer high potential in various applications due to their distinctive physicochemical, thermal, and mechanical properties [17,18]. Many drug formulations utilizing iron oxide NPs have received permission from the Food and Drug Administration (FDA) for commercial use, such as Ferinject^®^, Venofer^®^, and Ferrlecit^®^ for treating iron-deficient anaemia [19].

MRI offers state-of-the-art imaging solutions to radiologists and clinicians for proper diagnosis based on visualizing tissue morphology and anatomical features in both humans and animals. While MRI is a prominent imaging technique in oncology, its sensitivity is limited in the context of molecular and cellular imaging [20-22]. Magnetic contrast agents are often used to improve contrast and strengthen signals. While gadolinium diethylenetriaminopentaacetic acid (Gd-DTPA) is commonly used in clinical practice due to its strong *T*_1_ shortening effect, it has weak contrast enhancement and a rapid retention time *in vivo* [23,24]. Furthermore, the toxicity and biological safety of gadolinium during and after cellular endocytosis and its deposition in the brain remain largely unclear. Recent research indicates that magnetic iron oxide NPs are significantly more effective than Gd-DTPA as relaxation agents and possess advantageous magnetic behaviour [25-27]. Notably, iron oxide NPs also exhibit increased blood half-life, biodegradability, and non-toxic nature. Iron oxide NPs are indeed commonly used as *T*_2_ or negative CAs in MRI, making images darker. These *T*_2_ contrast agents negatively affect the pathological site due to their large magnetization values and magnetic behaviour [28]. Despite the contrasting ability and biocompatibility, iron oxide NPs based CAs are limited due to certain artefacts that may arise due to calcium deposits, internal bleeding, the presence of other metals, and signal voids present in the area being examined [29,30]. Due to these reasons, CAs such as Feridex^®^ and Endorem^®^ were discontinued, and positive contrast agents are much preferred in clinical settings, knowing their limitations [31].

Thus, there is an urgent need to develop non-toxic CAs, and in this context, several approaches have been adopted. Studies have shown that iron oxide NPs can be used to produce positive contrast in MRI by modifying the surface and the size [29,32]. Iron oxide NPs have been stabilized with functional groups to maintain stability and improve solubility. The surface coating can change the surface chemistry and electronic environment around Fe ions by affecting nearby water protons to relax through electronic exchange and influencing the local magnetic field fluctuations to enhance *T*_1_ relaxation. Biocompatible materials were mostly chosen as coating materials, depending on the type of application intended [33]. Typically, there are two primary methods for coating magnetic iron oxide NPs: in situ coatings, where the NPs are coated during the synthesis process, and post-synthesis coatings, applied after the NPs have been produced [34,35]. Carbon or silica shells provide a non-magnetic, chemically stable protective layer around MNPs, significantly enhancing their utility in various applications. Carbon has been demonstrated to offer greater stability against acids, bases, and oxidation compared to traditional polymer or silica shells [36]. Carbon-coated magnetic materials present a promising strategy for achieving enhanced chemical and mechanical stability, regardless of fluctuations in pH or temperature in the cellular microenvironment. This carbon shell with a magnetic core facilitates dipole-dipole interactions with water protons, enhancing *T*_1_ relaxation rather than *T*_2_ [37,38]. Moreover, the outer carbon shell of these MNPs can be readily modified with a variety of drugs and ligands, facilitating effective drug delivery and targeting of cancer cells [39]. While studies have investigated the impact of coating thickness on the relaxometry properties, the effect of the surface functional groups on the physicochemical properties of the iron oxide NPs remains largely unexplored [40]. The primary drawback of coating molecules on iron oxide NPs is the increase in particle size, often exceeding 100 nm following the coating process.

In this study, we present a simple one-pot hydrothermal synthesis procedure for the synthesis of variable degrees of carbon dot-coated iron oxide NPs. The relaxometry properties can be altered mainly by adjusting the composition of the precursors. Citric acid was chosen as the stabilizing agent for the synthesis of iron oxide NPs and also as a precursor for the formation of carbon dots [41]. The varying citric acid concentration will help to synthesize smaller-sized particles. Carbon coating can enhance the absorption intensities of MNPs in the visible light spectrum by creating oxygen defects on their surfaces [42,43]. Moreover, it reduces uniaxial anisotropy on the particle surface and lowers the average blocking temperature [44,45]. However, achieving stability and dispersion of the particles is often challenging due to their inadequate surface physicochemical properties. Carbon dots have several advantages, such as chemical inertness, high photostability, good solubility, tuneable surface, and photoluminescent properties. These features have made carbon dots a promising candidate for nanomedicine applications [46-48]. In this study, we hypothesized that the surface of iron oxide NPs can be modified with carbon dots to generate positive contrast. The effect of the surface modification on the physicochemical properties was also studied by varying the number of carboxylate molecules. The designed CDs@Fe_2_O_3_ can act as potential *T*_1_ and *T*_2_ CAs as well as an optical imaging fluorophore, which will enable the tracking of the path of the contrast agent *in vivo*. Thus, the fabricated NPs will serve as a biocompatible label-free multi-mode contrast agent.

## Materials and methods

Iron(II) chloride tetrahydrate (FeCl_2_.4H_2_O) and Iron (III) chloride hexahydrate (FeCl_3_.6H_2_O) were obtained from Sigma-Aldrich, India. Urea (CH_4_N_2_O), Citric acid (C_6_H_8_O_7_), XTT sodium salt (C_22_H_16_N_7_NaO_13_S_2_), and DMEM medium were purchased from Himedia Laboratories, India. All chemicals used were of analytical grade (≥99 %) and were utilized in their original form. Double-distilled water was employed for all experiments.

### Synthesis of carbon dot-coated iron oxide nanoparticles

One pot hydrothermal synthesis method was utilized to fabricate carbon dot-coated iron oxide nanoparticles (CDs@Fe_2_O_3_). The precursors used for the reaction are 1:2 molar ratios of 0.1M iron II and 0.2 M iron III chloride, 1 g urea, and various concentrations of citric acid added in 50 mL of double-distilled water. CDs@Fe_2_O_3_ were synthesized at five different concentrations, with varying concentrations of citric acid precursor from 1, 2, 3, 4 and 6 grams. The concentration of iron (II & III) chloride and urea did not change. The resultant samples were marked as C1, C2, C3, C4, and C5, respectively. The precursors were mixed and transferred to a Teflon-lined stainless-steel autoclave, which was maintained at 150 °C for 12 h, allowing the precursors to undergo a polymerization reaction. The reaction mixture was left to reach room temperature, and the polymer solution was purified by dialyzing against distilled water for 6 h in a dialysis membrane with 14 kDa MWCO (molecular weight cut-off). The resultant dispersant was then heated in a hot air oven to remove moisture at 150 °C for 10 h. The samples were then powdered and stored/used for further studies.

### Sample characterization

The crystalline nature of the CDs@Fe_2_O_3_ was investigated using a Smartlab Rigaku X-ray diffractometer, Japan. A Bruker-Alpha FTIR spectrometer (USA) was used to analyse the nature of chemical bonding. The X-ray photoelectron spectra (XPS) were recorded with VG Multilab 2000-Thermo Scientific K-alpha, USA. The magnetic nature of the samples was analysed using Lakeshore, USA VSM (vibrating sample magnetometer). Spectrometric characterization was done with a Shimadzu (Japan) UV-1800 spectrophotometer for absorption spectra, and a Jasco (FP-3800, Japan) Fluorimeter was used for fluorescent spectra. Surface morphology and size analysis of the NPs was carried out by Scanning electron microscope (SEM), JEOL JSM - 7600F, Japan. Particle size distribution and zeta potential analysis were performed using the Malvern, UK Nano-Zs particle size analyser. To perform *T*_1_ and *T*_2_ imaging, a GE, USA, Signa HDxT 1.5 T MRI scanner was used. Optical imaging was done using the Perkin-Elmer (USA) IVIS-Lumina LT *in vivo* imaging system. Surface area, pore size, and pore volume were studied using BETSORP Max BET Surface area analyser, Microtrac BEL, Japan.

### Haemolysis assay

The haemolysis assay was carried out following the established protocol from the literature, with slight modifications [36]. Fresh mice blood was collected in EDTA tubes, and red blood cells (RBCs) were collected by discarding the supernatant after centrifugation at 3000 rpm for 10 min. The RBCs were then washed five times with phosphate buffer saline (PBS). The purified RBCs were then diluted with PBS buffer 10 times. From the diluted RBCs, 100 μL was added into tubes containing 900 μL water (+ve control), 900 μL PBS (-ve control) and 9 μL PBS with sample concentrations ranging from 0.05 to 0.4 mg. Both control and samples were incubated at 37 °C for 2 h. After incubation, tubes were centrifuged at 12000 rpm for 2 min, and the absorbance (*A*) of the supernatants was measured at 541 nm to calculate the haemolysis, %, using Equation (1):



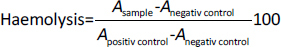

(1)


### Phantom magnetic resonance and fluorescence imaging

Phantom MRI was performed in a 96-well plate, where all five samples of CDs@Fe_2_O_3_ were added in varied concentrations of 10, 20, 30, 40, 60, 80 and 100 μg/mL. Following the standard protocols, both *T*_1_ and *T*_2_ weighted MR imaging were performed [49]. For *T*_1_ weighted imaging, the FLAIR sequence was opted to get a slice thickness of 2 mm with the parameters *T*_E_ (echo time) = 14 ms, *T*_R_ (repetition time) = 3000, FOV (field of view) = 24 24, and variable *T*_1_ was obtained from 400 to 2000 ms. *T*_2_ weighted imaging was performed using a turbo spin-echo sequence, acquiring 2 mm thick slices with a *T*_E_ range of 10-100 ms, *T*_R_ of 10000 ms, a field of view of 24 24 cm, and an echo train length of 12. The optical imaging ability of the CDs@Fe_2_O_3_ was validated *in vitro* using the IVIS small animal imaging system, PerkinElmer. *In vitro* imaging for sample C3 was carried out in a 96-well microtiter plate with increasing concentrations of 0, 15, 30, 45, 60, 75 and 90 μg/mL. The fluorescent images were acquired using a GFP filter at 460 nm.

### Cell viability assay

XTT assay was performed to analyse the cytotoxic effects of the synthesized CDs@Fe_2_O_3_ against FL (Follicular lymphoma) cells. In brief, 2×10^6^ cells were seeded per well in a 96-well plate and incubated at 37 °C, 5 % CO_2_ overnight. The cells were treated with 50, 100, 200, 300 and 400 mg/mL concentrations of CDs@Fe_2_O_3_ (Sample C3) for 24 h. After the incubation, the XTT reagent was added in dark conditions and then incubated for 5 h at 37 °C. DMSO was added and mixed well to dissolve the formazan crystals. The absorbance of the sample was measured with a microplate reader at 450 nm. The assay was done in triplicate.

### Hematology and histopathology

The animals were divided into 3 groups, each consisting of 6 mice. Group I was kept as control, while Group II and Group III were injected with the synthesized CDs@Fe_2_O_3_ (Sample C3). The animals in groups II and III were administered 500 μL of C3 through the tail vein, and the blood samples and organs were collected 1 h post-injection for group II and 24 h post-injection for group III. After a specified time, animals were anesthetized, the blood samples were collected by the retro-orbital method in EDTA-stabilized tubes, and the hematology assays were performed following the standard protocols [22]. A complete blood count, which includes measurements of red blood cells (RBC) and white blood cells (WBC), was performed using a modified Neubauer hemacytometer. The microhematocrit technique was utilized to determine the percentage of packed cell volume (PCV), and the concentration of haemoglobin was measured using the acid hematin method. Blood urea nitrogen (BUN) levels were obtained from serum samples analysed with semi-automatic analysers to evaluate the nephrological functions.

For histopathological analysis, body parts, including the heart, kidney, liver, and spleen, were harvested, and small pieces from them were taken and fixed in Bouin's solution and transferred to 10 vol.% formalin-neutral buffer [22]. Standard histological procedures have been utilized for tissue processing. After embedding with paraffin blocks, the organs were transversely sectioned to the desired thickness of 3 to 5 μm by using a rotatory microtome, and the sections were positioned on glass slides. The obtained sections were stained with H&E (Hematoxylin and eosin) for routine histopathological procedures and viewed under a light microscope at 4, 10, and 100a magnifications to investigate histoarchitecture.

### Ethical statement

All the animal experiments were conducted following the ethical guidelines of the Institutional Animal Ethics Committee (IAEC) of Chettinad Hospital and Research Institute (CHRI), Chettinad Academy of Research and Education (CARE). The reference number was IAEC3/Proposal: 27/A.Lr: 09. The IAEC approved all the experimental protocols related to the study.

## Results and discussion

### Physicochemical characterizations

**Figure 1.**
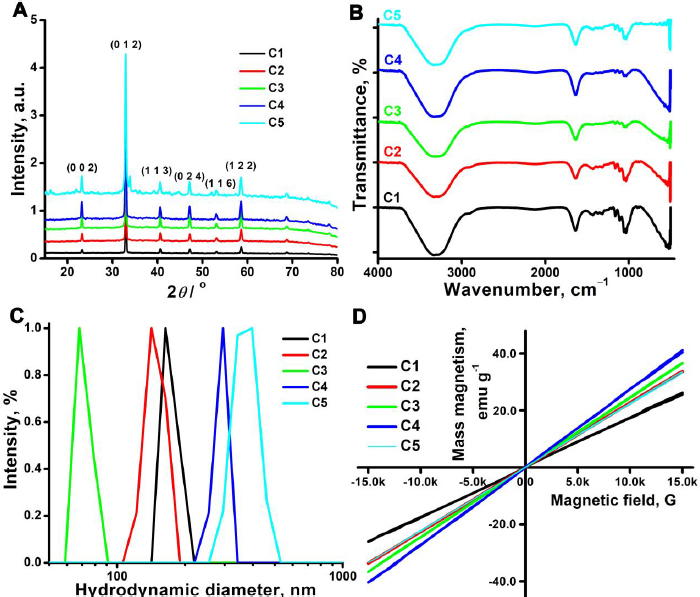
A) XRD pattern of CDs@Fe_2_O_3_; B) FTIR spectra of CDs@Fe_2_O_3_; C) Particle size distribution of CDs@Fe_2_O_3_; D) VSM spectra of CDs@Fe_2_O_3_

The crystalline structure and phase identification of the synthesized samples were investigated by powder X-ray diffraction technique (Figure 1A) [50]. The purity of the samples was confirmed by the strong and sharp Bragg's reflections assigned to (104), (113), (116), (018) and (208) planes of the Miller indices, respectively (PDF # 33-0664). The diffraction peaks can be indexed to pure rhombohedral α-Fe_2_O_3_ NPs [51]. Further, the peak cantered at 23.06° is attributed to the highly disordered carbon atoms, thereby confirming the presence of the amorphous carbon shell over the iron oxide core. The XRD peaks correspond well with the standard XRD pattern for α-Fe_2_O_3_ (hematite) (JCPDS card no 87-1166), confirming that synthesized iron oxide NPs are hematite in nature. No phase change was observed among the samples, and all the samples showed the same peak positions, as shown in Figure 1A. By utilizing the Scherrer equation, it was possible to determine the crystallite size of CDs@Fe_2_O_3_. The calculated crystallite sizes of the samples are represented in Table 1. It was observed that the variation in the amount of citric acid has a considerable impact on the size of the NPs. Citric acid added to the reaction mixture not only acts as a precursor for the formation of carbon dots but also acts as a reducing agent in the synthesis of iron oxide NPs. The C3 sample shows a smaller crystalline size compared to other samples.

**Table 1.** Comparison of hydrodynamic diameter, zeta potential, crystalline size and saturation magnetization for the samples C1 to C5

CDs@Fe_2_O_3_	Hydrodynamic diameter, nm	Zeta potential, mV	Crystalline size, nm	Saturation magnetization, emu g^-1^
C1	164±3	-32±2	103±4	16.84
C2	143±4	-53±3	61±2	20.42
C3	68±7	-78±2	55±1	24.14
C4	294±3	-48±4	62±2	24.56
C5	369±5	-10±2	118±3	19.48

The functional groups and chemical bonding nature of the samples were analysed using FTIR, as shown in Figure 1B. The broad absorption band at 3327 cm^-1^ corresponds to the O-H stretching vibration of carboxylic acid [52]. The carboxylic functional groups impart a hydrophilic nature and enhance the stability of the CDs@Fe_2_O_3_. The asymmetric and symmetric sharp bands at 1638 cm^-1^ and 1041 cm^-1^ correspond to the N-H bending and C-N stretching of amine. The peaks observed at 1160, 1437 and 1635 cm^-1^ correspond to C-C, C-O and C=C bond vibrations of carbon. The absorption band at 534 cm^-1^ indicates the presence of the Fe-O bond of the iron oxide NPs. All of the obtained FTIR bands revealed the formation of carbon dots and iron oxide NPs, and no notable differences were seen in the absorption bands of the samples from C1-C5 (Figure 1B) [51].

A dynamic light scattering technique was applied to determine the hydrodynamic diameter (dH) and zeta potential of the synthesized CDs@Fe_2_O_3_, and the results are exhibited in Table 1. The recorded hydrodynamic diameters of the samples C1-C5 are represented in Figure 1C. The increasing concentration of citric acid typically leads to the formation of smaller carbon dots due to its increased nucleation rates. Lower (1 and 2 g) citric acid concentration makes larger particles due to incomplete reaction with urea and poor stabilization. 3 g citric acid shows the smallest NPs, and good surface functionalization is due to sufficient carbon formation and uniform coating. The higher concentrations of 4 and 6 g of citric acid produce particles of larger sizes due to their inappropriate carbon masking, making a thicker shell. From the results, C3 is certainly the optimal value of citric acid to be added to synthesize the lowest possible dH of the CDs@Fe_2_O_3_. Changes in concentrations (higher or lower) will result in the synthesis of particles with higher dH. Furthermore, zeta potential results also support the idea that sample C3 has the highest particle stability in comparison to the other samples (Table 1).

With the brief discussions about the effect of the surface chemistry on the size and stability of the CDs@Fe_2_O_3_, we speculated that the good affinity of the carboxylate molecules to adsorb on the iron oxide surface would also ideally alter the saturation magnetization (Ms) values of the samples. To support this, we investigated the Ms values of the CDs@Fe_2_O_3_ at room temperature by employing vibrating sample magnetometry (VSM). The plotted VSM spectra of the samples are presented in Figure 1D. The obtained Ms values are less than those of the commercially available iron oxide NPs. The Ms value of the sample C3 is slightly higher than the other samples (Table 1). This variation in the Ms values can be attributed to the canted spins in the oxidized layer of the iron oxide NPs. Magnetic particles in the nanoscale tend to act as a core-shell material, with the core composed of aligned spins and the latter composed of disordered spins. The layer with the disordered spins is also known as the dead layer or oxidized layer. These disordered spins can interact ferromagnetically with the external magnetic field and can contribute to the total magnetization value. As evident from the stability values of the Zeta potential, C3 has plenty of carboxylate molecules, which also means that the surface spin canting will be higher in C3 than in the other particles. The Ms values of the other samples are lower than C3, as they are larger and do not have a high amount of carboxylate molecules on their surface, as C3.

### Surface chemistry and morphology

**Figure 2:**
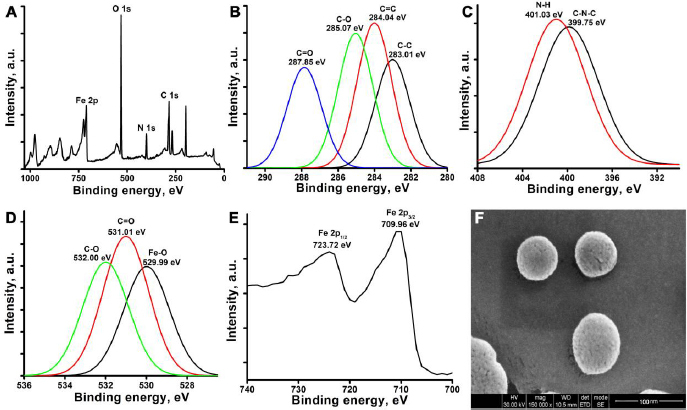
A) XPS image of CDs@Fe_2_O_3_ (C3) to verify the structure and chemical composition. Fitted narrow range XPS spectra of B) C 1s, C) N 1s, D) O 1s, and E) Fe 2p of CDs@Fe_2_O_3_ (C3). F) SEM image CDs@Fe_2_O_3_ (C3)

XPS was employed to ascertain the chemical composition, oxidation states, and bonding of the C3. The full range XPS spectrum presented in Figure 2A represents the photoelectron peaks of Fe 2p, C 1s, O 1s, and N 1s at 709.96, 284.04, 531.01 and 399.75 eV, respectively.

The high-resolution spectra of each element are presented in Figure 2B-E. The binding energies at 723.72 and 709.96 eV correspond to Fe 2p_1/2_ and Fe 2p_3/2_. In the O 1s spectrum, the photoelectron line at 529.99 eV binding energy corresponds to the Fe-O bonding, and the binding energy at 531.01 and 532.00 eV is attributed to C=O and C-O. From the Fe 2p and O 1s, it can be deduced that the valence state of the iron oxide core is +3 and -2, respectively. This affirms that the iron oxide is in the hematite phase, and there is no sign of impurity or phase transformation [51]. The high-resolution spectra of the C 1s spectra exhibit four peaks at 284.04 eV (C=C), 283.01 eV (C-C), 285.07 eV (C-O), 287.85 eV (C=O). The peaks at 284.04 and 283.01 eV binding energies correspond to the C sp2 and C sp3 peaks of graphitized and amorphous carbon. The N 1s spectra have two peaks at 399.75 and 401.03 eV, which represent the C-N-C and the amine groups (N-H) present at the surface of the carbon dots and also support the formation of CDs@Fe_2_O_3_ [51]. The obtained XPS results are in line with the results obtained from XRD and FTIR studies. The morphology of the C3, as observed in the SEM, is shown in Figure 2F. The SEM image showed that particles of uniform spherical structure are homogeneously distributed. The mean diameter of the C3 was calculated as 55 ± 4.5 nm.

### Surface area analysis

**Figure 3.**
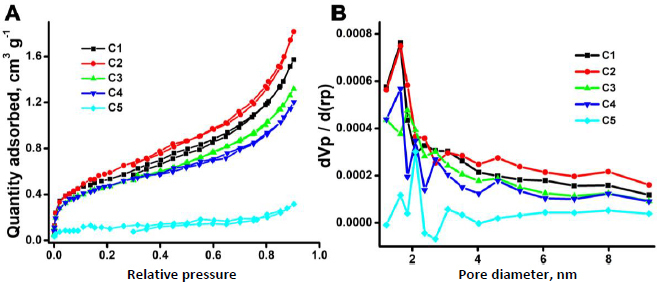
BET analysis of CDs@Fe_2_O_3_: A) Nitrogen adsorption/desorption isotherm plots and B) BJH plots of the pore size distribution

The synthesized CDs@Fe_2_O_3_ (C1-C5) surface area was investigated using the Brunauer-Emmett-Teller method (BET) (Figure 3A). The mean pore diameter, pore volume, and surface area of the samples are summarized in Table 2. To understand the porous characteristic of the samples, nitrogen gas adsorption-desorption isotherms were obtained for all the samples, and the plots were identified as type IVa isotherms having an H1 hysteresis loop according to the IUPAC standard [53]. The type IVa isotherm suggests the presence of inhomogeneous mesopores in samples, which is additionally verified by the Barrett, Joyner, and Halenda (BJH) pore size distribution plots (Figure 3B). The BJH method confirmed the existence of mesopores, revealing that the pore diameters of the samples are between 2 and 50 nm. From Table 2, it can be observed that the samples are mesoporous, with different pore diameters and volumes, because of the variation in the concentration of citric acid. As already outlined in DLS and XRD, the samples are of different sizes; hence, the surface area characteristics have also differed, confirming the initial observations.

**Table 2.** Mean pore diameter, total pore and mesopore volume and surface area of CDs@Fe_2_O_3_ obtained by BET analysis

CDs@Fe_2_O_3_	Mean pore diameter, nm	Total pore volume, cm^3^ g^-1^	Mesopore volume, cm^3^ g)	BET surface area, m^2^ g^-1^
C1	5.15	0.0028	0.4339	1.89
C2	5.06	0.0024	0.5099	2.22
C3	4.84	0.0020	0.3870	1.68
C4	4.32	0.0018	0.3961	1.72
C5	4.85	0.0009	0.0926	0.40

### Spectrophotometric and fluorometric analysis

**Figure 4.**
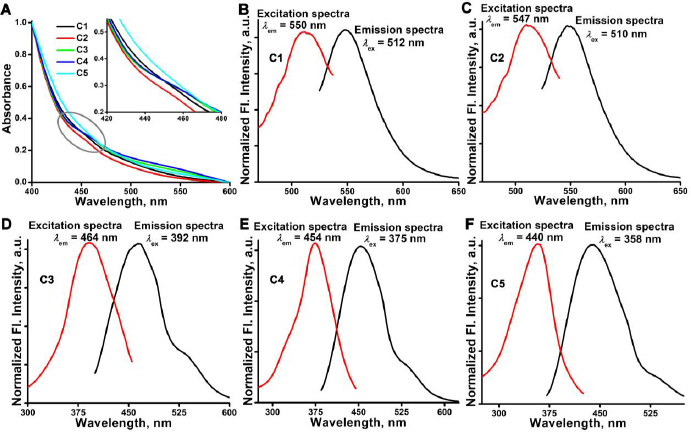
A) Absorption spectra of CDs@Fe_2_O_3_ (Inset is the magnified part of the absorption spectra). Steady-state fluorescence spectra (emission and excitation) of CDs@Fe_2_O_3_: B) C1, C) C2, D) C3, E) C4 and F) C5

The synthesized CDs@Fe_2_O_3_ showed a broad absorption band ranging from 430 to 465 nm centered at 452 nm, depicting the formation of the carbon dots around the core of the MNPs as represented in Figure 4A. A similar absorption peak was observed for all the samples (C1-C5). The obtained absorption range is solely attributed to the presence of carbon dots because neither citric acid nor urea absorbs within the wavelength range [54,55].

The broad absorption peak was further analysed by steady-state fluorescence measurement to find out the emission maxima, followed by excitation spectra to get the exact excitation wavelength of C1 to C5. Interestingly, the emission maxima (*λ*_em_^max^) were 550 nm for C1 and showed a continuous reduction of 547 nm for C2, 464 nm for C3, 454 nm for C4 and 440 nm for C5 (Figures 4B to 4F). A similar shift was observed when excitation spectra were recorded fixing *λ*_em_^max^ at their (C1-C5) individual maxima. A narrow and sharp excitation peak was observed at 512 nm for C1, 510 nm for C2, 392 nm for C3, 375 nm for C4, and 358 nm for C5 (Figure 4B to 4F). Increasing the concentration of citric acid, keeping the concentration of Fe(II), Fe(III), and urea fixed in the synthesis, can modify the surface chemistry of the fluorescent particles, enhance surface passivation or altering the types of functional groups presented on their surface. This alteration can impact the electronic environment, resulting in a shift in the fluorescent wavelength. Urea functions as the source of carbon, along with citric acid and amino functional groups that contribute to the fluorescence properties. It was reported that urea and citric acid are involved in an open and closed reaction system during the hydrothermal process to produce a polymer-like structure that occupies the surface of the magnetic cores [49]. This peak shift confirms the binding mechanism of carbon dots on the surface of MNPs [49]. The fluorescence imaging capability of C3 was further analysed by the IVIS optical imaging system.

### Phantom magnetic resonance imaging

**Figure 5.**
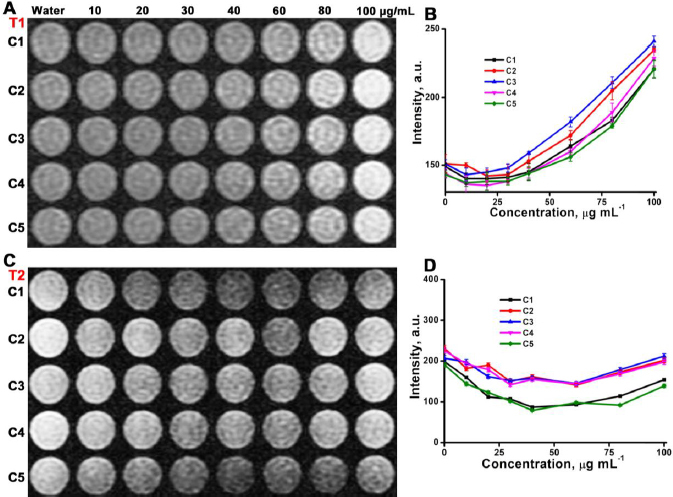
A) *T*_1_ weighted phantom images of CDs@Fe_2_O_3_ (C1-C5). B) Plotting the intensities from *T*_1_ weighted images against increasing concentrations provides insights into CDs@Fe_2_O_3_ (C1-C5). C) *T*_2_ weighted phantom images of CDs@Fe_2_O_3_ (C1-C5). D) Plotting the intensities from *T*_2_ weighted images against increasing concentrations provides insights into CDs@Fe_2_O_3_ (C1-C5)

A phantom MRI study was executed to evaluate the contrasting ability of the CDs@Fe_2_O_3_ (Figure 5). The samples were prepared in different concentrations and then imaged under the MRI scanner following the standard protocols. The obtained images were analysed with the DICOM image processing program supplied by the manufacturer, demonstrating that the samples exhibited good positive contrast in *T*_1_ weighted MRI and negative contrast in the *T*_2_ weighted images. The carbon coating acts as a barrier between Fe_2_O_3_ NPs, preventing agglomeration. Dispersed particles, with minimal clustering, exhibit reduced magnetic inhomogeneity, allowing them to contribute to *T*_1_ (longitudinal) relaxation more effectively. The image intensities significantly rise in *T*_1_ weighted images for increasing concentration and moderately decrease in *T*_2_ weighted images for limited concentration (Figure 5A to 5D). The C3 sample shows better *T*_1_ relaxivity than other samples and the brightening effect started to increase sharply after 30 μg ml^-1^ concentration (Figure 5A to 5B). The *T*_2_ intensity is almost similar for C2-C4 (Figure 5C-D) and slightly higher than the other two samples, C1 and C5. Therefore, the synthesized CDs@Fe_2_O_3_ show a prominent *T*_1_ effect rather than *T*_2_. Evaluating the contrasting ability of CDs@Fe_2_O_3_ requires determining the longitudinal (*r*_1_ = 1/*T*_1_) and transverse (*r*_2_ = 1/*T*_2_) relaxivity values. These values were derived from *T*_1_ FLAIR images with varying TI ranging from 400 to 2000ms and *T*_2_ TSE images with TE ranging from 10 to 100 ms. Graphs were plotted to show the *T*_1_ and *T*_2_ intensities in relation to the increasing concentrations of CDs@Fe_2_O_3_, assessing both molar relaxivities (Figure 5B). The *r*_1_ molar relaxivities for C1, C2, C3, C4 and C5 were calculated as 4.1±0.8, 5.9±0.4, 6.2±1.0, 5.1±0.5 and 3.8±0.7 mM^-1^ s^-1^, respectively. The *r*_2_ values were calculated for C1, C2, C3, C4, and C5 as 29.3±1.9, 31.2±1.1, 32.1±1.1, 32.8±1.6 and 28.9±1.0 mM^-1^ s^-1^, respectively. The calculated data show better relaxivities compared to commercially available contrast agent Gd-DOTA (*r*_1_ and *r*_2_ relaxivities of 2.94 and 3.60 mM^−1^ s^−1^, respecttively), as reported by Thirumalai *et al.* [47]. The *r*_2_/*r*_1_ ratio for sample C1-C5 lies in the range of 10 > *r*_2_/*r*_1_ > 3.

The relaxivity results show that CDs@Fe_2_O_3_ has enhanced *T*_1_ relaxation rather than *T*_2_, as observed earlier in the intensity. This is due to carbon coating influencing the relaxation of nearby water protons through the electronic exchange, affecting the local magnetic field fluctuations, favouring *T*_2_ relaxation by facilitating dipole-dipole interactions with water protons. This indicates the potential of the CDs@Fe_2_O_3_ to act as an efficient twin-mode *T*_1_ and *T*_2_ CAs in MR imaging, and sample C3 shows higher *T*_1_ signal intensity than the other samples. The C3 features an optimal CD coating that is thin enough to maintain water access to the iron core, hydrophilic enough to attract water, and stable enough to prevent aggregation. This ideal combination leads to improved *T*_1_ relaxivity. This observation follows those obtained from the zeta potential analysis, hydrodynamic diameter, XRD patterns and VSM. Hence, sample C3 was used for further experiments.

### Phantom optical imaging

**Figure 6.**
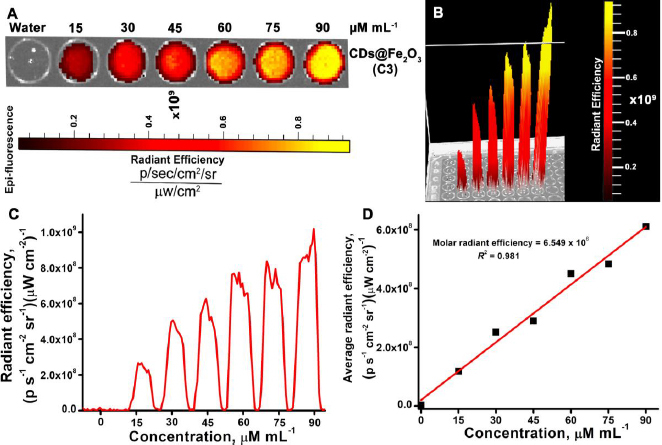
A) IVIS-based fluorescent phantom images of CDs@Fe_2_O_3_ (C3) at increasing concentration. B) 3D image of the phantom to show the change in fluorescent intensities in each well. C) Radiant efficiencies of C3 were plotted against the concentration. D) Fluorescent intensities were plotted against concentration to calculate the average radiant efficiencies

The fluorescence ability of the C3 was assessed by phantom optical imaging. Sample C3 was taken in increasing concentrations (0, 15, 30, 45, 60, 75 and 90 μM ml^-1^) and imaged in a small animal imaging system, as shown in Figure 6. It was observed that the fluorescence intensity gradually increased with the higher concentration of C3 (Figure 6A-C). The images obtained from optical imaging were adjusted to reflect photons *per* second *per* square centimetre *per* steradian (*p* s^-1^ cm^-2^ sr^-1^) following the elimination of background signals. A gradual increase in intensity was noted with rising concentrations of C3 of CDs@Fe_2_O_3_. Figure 6D illustrates the graphical depiction of average radiant efficiency alongside the concentrations of CDs@Fe_2_O_3_, where the slope indicates the average radiant efficiency of 6.549 × 10^6^ p/sec/cm^2^/sr per mM. The data clearly showed that the CDs@Fe_2_O_3_ (sample C3) can be used for multimodal imaging.

### In vitro and in vivo toxicity assessment

**Figure 7.**
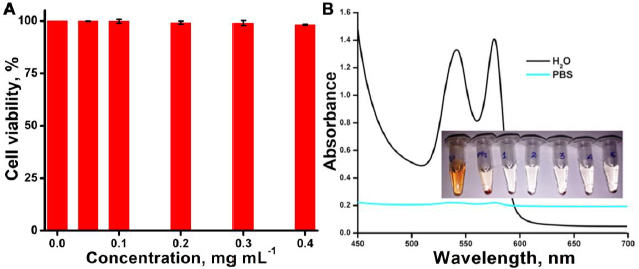
A) XTT assay using FL cells to evaluate the toxicity of CDs@Fe_2_O_3_ (C3). B) Evaluation of hemocompatibility of CDs@Fe_2_O_3_ (C3) at varying concentrations (Inset: photograph of the samples incubated with variable concentrations of CDs@Fe_2_O_3_)

*In vitro*, cytotoxicity assessment of synthesized CDs@Fe_2_O_3_ (Sample C3) was evaluated by XTT cell viability assay performed on FL cell lines. The obtained data shows that the synthesized particles show similar cell viability compared to the control (Figure 7A). Sample C3 does not exhibit significant toxicity, even at the highest concentration. As a result, it was determined that the toxic effect of ferrite nanoparticles was significantly mitigated by the carbon coating, indicating that the synthesized CDs@Fe_2_O_3_ (Sample C3) are biocompatible. A haemolysis assay is essential to investigate the hemocompatibility of the prepared NPs and ensure *in vivo* safety. For the haemolysis assay, the CDs@Fe_2_O_3_ (Sample C3) was taken in five different concentrations ranging from 0.05, 0.1, 0.2, 0.3 and 0.4 mg/ml. Based on the absorbance values, the percentage of haemolysis was calculated, and it was found to be <2 % for the sample with a high concentration of the CDs@Fe_2_O_3_. This value shows that the synthesized particle is haemocompatible and complies with the ASTM E2524-08 standard parameter (Standard test method for analysis of hemolytic products of NPs). As per the ASTM standards, NPs that show less than 5 % heamolytic activity are considered haemocompatible. The absorbance spectrum of the positive control and negative control, along with the photograph of the haemolysis activity, is represented in Figure 7B. The haemolysis percentage obtained for the CDs@Fe_2_O_3_ (C3) aligns well with the standard value, emphasizing the remarkable hemocompatibility of the CDs@Fe_2_O_3_.

Haematological analysis is an essential method for evaluating the toxic effects of nanomaterials by studying their interactions with blood and its compounds. The hematologic parameters, such as packed cell volume (PCV), RBC, WBC, and haemoglobin were investigated from the whole blood. The nephrotoxicity of the administered NPs was assessed by monitoring the BUN values in serum samples collected from animal models divided into 3 groups. The obtained results are shown in Table 3. A moderate decrease in RBC count after 1 h of injection, which increased to normal after 24 h of injection, was noted, and the PCV was also increased for 1 h of injection. The decrease in RBC level is due to the higher oxygen level and decreased haemoglobin metabolism regulated by erythropoietin secretion in the kidney. The leukocyte count was recorded in all three groups and showed an increase over time, indicating an enhanced immune response to the injected foreign material. However, despite the increase in WBC count, it is deemed insignificant as the levels remained low and within the normal range. This suggests that the majority of the injected CDs@Fe_2_O_3_ are able to evade the immune response.

**Table 3.** Haematological parameters were assessed in mice with and without injection of CDs@Fe_2_O_3_ (C3)

	Control	After 1 hour	After 24 hour	Reference values
Number of WBC, 10^3^ mm^3^	9.80	10.9	12.3	7.20 to 12.60
Number of RBC, 10^6^ mm^3^	9.50	6.89	9.66	7.21 to 8.45
Haemoglobin concentration, g/dl	14.6	11.1	15.5	13.2 to 16.4
PCV, %	43.8	56.1	47.1	43.6 to 48.6
BUN concentration, mg/dl	21.9	39.71	28.50	10 to 33.00

Kidneys play a major role in renal filtration, and the results show that the BUN values increase after 1 h and return to normal after 24 h. Thus, the BUN evaluation shows that the metabolism of Fe_2_O_3_NPs in the kidneys depends on time. Even though there were slight variations in the hematologic values, all the values were in the normal range.

**Figure 8.**
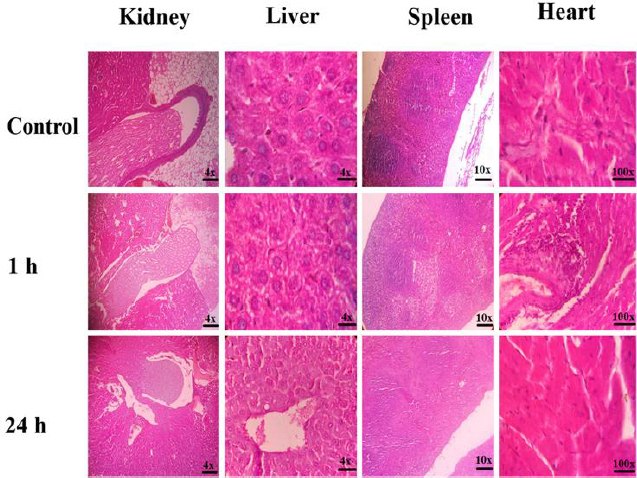
Histopathological analyses of kidney, liver, spleen, and heart of mice injected with CDs@Fe_2_O_3_ (C3)

Histopathological examination of the mice was conducted to analyse the impact of the CDs@Fe_2_O_3_ (C3) on different organs, utilizing Haematoxylin and Eosin (H&E) staining, as illustrated in Figure 8. No significant changes were noted between the control group and the other two groups administered with CDs@Fe_2_O_3_. From the photomicrographs of the liver and kidney, it can be seen that the architecture of the liver and kidney in the treatment groups is similar to that of the reference group. In the liver, the hepatocytes exhibit clear cytoplasm, while in the kidney, the papilla, inner medulla, and cortex appear normal. No abnormal lesions were found in the heart tissues, and no changes were observed in the red pulp or white pulp of the spleen. Thus, it can be inferred that the administration of the C3 sample of CDs@Fe_2_O_3_ doesn't result in any significant changes in the mice organs investigated and thus is safe to be administered *in vivo*.

## Conclusion

In summary, this study has developed a promising method for synthesizing carbon dot-coated iron oxide nanoparticles (CDs@Fe_2_O_3_) and standardizing the concentration of citric acid that regulates both the reaction process and the size of the iron oxide nanocluster. It was identified that changes in the surface chemistry of iron oxide NPs can have considerable effects on the physicochemical properties. The characteristic properties of the synthesized CDs@Fe_2_O_3_ highlight the stability attained through the specific concentration of citric acid employed in this synthesis method. Based on the findings from phantom imaging, it is clear that the CDs@Fe_2_O_3_ exhibit superior magnetic and fluorescent properties, making them suitable for use as an effective dual-mode MR imaging agent in conjunction with fluorescent imaging. The concentration of precursors to obtain maximum contrast enhancement has been optimized, and the same has been confirmed with the findings from characterization studies such as FTIR, XRD, VSM, zeta potential analysis, and DLS measurements. The synthesized particles were found to be negligibly toxic and possess good hemocompatibility. The present study offers insights into the biocompatibility of the CDs@Fe_2_O_3_ (C3) synthesized via a robust method. This was assessed by evaluating haematological parameters and kidney function through BUN levels and conducting histopathological analyses of various organs in mice. Thus, the synthesized CDs@Fe_2_O_3_ shows promising results for being considered as an alternative biocompatible multimodal contrast agent.

## References

[1] Fernandes D.A.. Review on metal-based theranostic nanoparticles for cancer therapy and imaging. Technology in Cancer Research & Treatment 22 (2023) 15330338231191493. https://doi.org/10.1177/15330338231191493 10.1177/15330338231191493 37642945 PMC10467409

[2] Fernandes D.A.. Review on iron nanoparticles for cancer theranostics: synthesis, modification, characterization and applications. Journal of Nanoparticle Research 25 (2023) 170. https://doi.org/10.1007/s11051-023-05807-1 10.1007/s11051-023-05807-1

[3] Girigoswami K. Pallavi P. Girigoswami A.. Intricate subcellular journey of nanoparticles to the enigmatic domains of endoplasmic reticulum. Drug Delivery 30 (2023) 2284684. https://doi.org/10.1080/10717544.2023.2284684 10.1080/10717544.2023.2284684 37990530 PMC10987057

[4] Elumalai K. Srinivasan S. Shanmugam A.. Review of the efficacy of nanoparticle-based drug delivery systems for cancer treatment. Biomedical Technology 5 (2024) 109-122. https://doi.org/10.1016/j.bmt.2023.09.001 10.1016/j.bmt.2023.09.001

[5] Harini K. Girigoswami K. Pallavi P. Gowtham P. Thirumalai A. Charulekha K. Girigoswami A.. MoS2 nanocomposites for biomolecular sensing, disease monitoring, and therapeutic applications. Nano Futures 7 (2023) 032001. https://doi.org/10.1088/2399-1984/ace178 10.1088/2399-1984/ace178

[6] Vedakumari S.W. Senthil R. Sekar S. Babu C.S. Sastry T.P.. Enhancing anti-cancer activity of erlotinib by antibody conjugated nanofibrin - In vitro studies on lung adenocarcinoma cell lines. Materials Chemistry and Physics 224 (2019) 328-333. https://doi.org/10.1016/j.matchemphys.2018.11.061 10.1016/j.matchemphys.2018.11.061

[7] Affrald R J. M N. Narayan S.. A comprehensive review of manganese dioxide nanoparticles and strategy to overcome toxicity. Nanomedicine Journal 10 (2023) 1-15. https://doi.org/10.22038/nmj.2022.66131.1694 10.22038/nmj.2022.66131.1694

[8] Wu K. Su D. Liu J. Saha R. Wang J.-P.. Magnetic nanoparticles in nanomedicine: a review of recent advances. Nanotechnology 30 (2019) 502003. https://doi.org/10.1088/1361-6528/ab4241 10.1088/1361-6528/ab4241 31491782

[9] Anik M.I. Hossain M.K. Hossain I. Mahfuz A.M.U.B. Rahman M.T. Ahmed I.. Recent progress of magnetic nanoparticles in biomedical applications: A review. Nano Select 2 (2021) 1146-1186. https://doi.org/10.1002/nano.202000162 10.1002/nano.202000162

[10] Khizar S. Ahmad N.M. Zine N. Jaffrezic-Renault N. Errachid-el-salhi A. Elaissari A.. Magnetic Nanoparticles: From Synthesis to Theranostic Applications. ACS Applied Nano Materials 4 (2021) 4284-4306. https://doi.org/10.1021/acsanm.1c00852 10.1021/acsanm.1c00852

[11] Harini K. Girigoswami K. Pallavi P. Gowtham P. Prabhu A.D. Girigoswami A.. Advancement of magnetic particle imaging in diagnosis and therapy. Advances in Natural Sciences: Nanoscience and Nanotechnology 15 (2024) 023002. https://doi.org/10.1088/2043-6262/ad3b7a 10.1088/2043-6262/ad3b7a

[12] Comanescu C.. Magnetic Nanoparticles: Current Advances in Nanomedicine, Drug Delivery and MRI. Chemistry 4 (2022) 872-930. https://doi.org/10.3390/chemistry4030063 10.3390/chemistry4030063

[13] Dubey R. Sinha N. Jagannathan N.R.. Potential of in vitro nuclear magnetic resonance of biofluids and tissues in clinical research. NMR in Biomedicine 36 (2023) e4686. https://doi.org/10.1002/nbm.4686 10.1002/nbm.4686 34970810

[14] Haribabu V. Girigoswami K. Girigoswami A.. Magneto-silver core–shell nanohybrids for theragnosis. Nano-Structures & Nano-Objects 25 (2021) 100636. https://doi.org/10.1016/j.nanoso.2020.100636 10.1016/j.nanoso.2020.100636

[15] Joga S.B. Korabandi D. Lakkaboyana S.K. Kumar V.. Synthesis of iron nanoparticles on lemon peel carbon dots (LP-CDs@Fe3O4) applied in Photo-Catalysis, Antioxidant, Antidiabetic, and Hemolytic activity. Inorganic Chemistry Communications 174 (2025) 113960. https://doi.org/10.1016/j.inoche.2025.113960 10.1016/j.inoche.2025.113960

[16] Perumalsamy H. Balusamy S.R. Sukweenadhi J. Nag S. MubarakAli D. El-Agamy Farh M. Vijay H. Rahimi S.. A comprehensive review on Moringa oleifera nanoparticles: importance of polyphenols in nanoparticle synthesis, nanoparticle efficacy and their applications. Journal of nanobiotechnology 22 (2024) 71. https://doi.org/10.1186/s12951-024-02332-8 10.1186/s12951-024-02332-8 38373982 PMC10877787

[17] Kumar S. Kumar M. Singh A.. Synthesis and characterization of iron oxide nanoparticles (Fe2O3, Fe3O4): a brief review. Contemporary Physics 62 (2021) 144-164. https://doi.org/10.1080/00107514.2022.2080910 10.1080/00107514.2022.2080910

[18] Farinha P. Coelho J.M.P. Reis C.P. Gaspar M.M.. A Comprehensive Updated Review on Magnetic Nanoparticles in Diagnostics. Nanomaterials 11 (2021) 3432. https://doi.org/10.3390/nano11123432 10.3390/nano11123432 34947781 PMC8706278

[19] Anselmo A.C. Mitragotri S.. Nanoparticles in the clinic. Bioengineering & translational medicine 1 (2016) 10-29. https://doi.org/10.1002/btm2.10003 10.1002/btm2.10003 29313004 PMC5689513

[20] Chauhan P. Kushwaha P.. Applications of Iron Oxide Nanoparticles in Magnetic Resonance Imaging (MRI). Nanoscience & Nanotechnology-Asia 11 (2021) 290-299. https://doi.org/10.2174/2210681210999200728102036 10.2174/2210681210999200728102036

[21] Dwivedi D.K. Jagannathan N.R.. Emerging MR methods for improved diagnosis of prostate cancer by multiparametric MRI. Magnetic Resonance Materials in Physics, Biology and Medicine 35 (2022) 587-608. https://doi.org/10.1007/s10334-022-01031-5 10.1007/s10334-022-01031-5 35867236

[22] Haribabu V. Sharmiladevi P. Akhtar N. Farook A.S. Girigoswami K. Girigoswami A.. Label Free Ultrasmall Fluoromagnetic Ferrite-clusters for Targeted Cancer Imaging and Drug Delivery. Current Drug Delivery 16 (2019) 233-241. https://doi.org/10.2174/1567201816666181119112410 10.2174/1567201816666181119112410 30451110

[23] Iyad N. Ahmad M. S. Alkhatib S.G. Hjouj M.. Gadolinium contrast agents- challenges and opportunities of a multidisciplinary approach: Literature review. European Journal of Radiology Open 11 (2023) 100503. https://doi.org/10.1016/j.ejro.2023.100503 10.1016/j.ejro.2023.100503 37456927 PMC10344828

[24] Murugesan B. Ramanarayanan S. Vijayarangan S. Ram K. Jagannathan N.R. Sivaprakasam M.. A deep cascade of ensemble of dual domain networks with gradient-based *T*_1_ assistance and perceptual refinement for fast MRI reconstruction. Computerized medical imaging and graphics 91 (2021) 101942. https://doi.org/10.1016/j.compmedimag.2021.101942 10.1016/j.compmedimag.2021.101942 34087612

[25] Gowtham P. Girigoswami K. Prabhu A.D. Pallavi P. Thirumalai A. Harini K. Girigoswami A.. Hydrogels of Alginate Derivative-Encased Nanodots Featuring Carbon-Coated Manganese Ferrite Cores with Gold Shells to Offer Antiangiogenesis with Multimodal Imaging-Based Theranostics. Advanced Therapeutics 7 (2024) 2400054. https://doi.org/10.1002/adtp.202400054 10.1002/adtp.202400054

[26] Eguía-Eguía S.I. Gildo-Ortiz L. Pérez-González M. Tomas S.A. Arenas-Alatorre J.A. Santoyo-Salazar J.. Magnetic domains orientation in (Fe3O4/γ-Fe2O3) nanoparticles coated by Gadolinium-diethylenetriaminepentaacetic acid (Gd3+-DTPA). Nano Express 2 (2021) 020019. https://doi.org/10.1088/2632-959X/ac0107 10.1088/2632-959X/ac0107

[27] Yano K. Matsumoto T. Okamoto Y. Kurokawa N. Hasebe T. Hotta A.. Fabrication of Gd-DOTA-functionalized carboxylated nanodiamonds for selective MR imaging (MRI) of the lymphatic system. Nanotechnology 32 (2021) 235102. https://doi.org/10.1088/1361-6528/abeb9c 10.1088/1361-6528/abeb9c 33657547

[28] Frantellizzi V. Conte M. Pontico M. Pani A. Pani R. De Vincentis G.. New Frontiers in Molecular Imaging with Superparamagnetic Iron Oxide Nanoparticles (SPIONs): Efficacy, Toxicity, and Future Applications. Nuclear Medicine and Molecular Imaging 54 (2020) 65-80. https://doi.org/10.1007/s13139-020-00635-w 10.1007/s13139-020-00635-w 32377258 PMC7198685

[29] Bao Y. Sherwood J. Sun Z.. Magnetic iron oxide nanoparticles as T 1 contrast agents for magnetic resonance imaging. Journal of Materials Chemistry C 6 (2018) 1280-1290. https://doi.org/10.1039/C7TC05854C 10.1039/C7TC05854C

[30] Kothandaraman H. Kaliyamoorthy A. Rajaram A. Kalaiselvan C.R. Sahu N.K. Govindasamy P. Rajaram M.. Functionalization and Haemolytic analysis of pure superparamagnetic magnetite nanoparticle for hyperthermia application. Journal of Biological Physics 48 (2022) 383-397. https://doi.org/10.1007/s10867-022-09614-y 10.1007/s10867-022-09614-y 36434309 PMC9727058

[31] Wang Y.-X.J.. Current status of superparamagnetic iron oxide contrast agents for liver magnetic resonance imaging. World journal of gastroenterology 21 (2015) 13400. https://doi.org/10.3748/wjg.v21.i47.13400 10.3748/wjg.v21.i47.13400 26715826 PMC4679775

[32] Fernández-Barahona I. Muñoz-Hernando M. Ruiz-Cabello J. Herranz F. Pellico J.. Iron Oxide Nanoparticles: An Alternative for Positive Contrast in Magnetic Resonance Imaging. Inorganics 8 (2020) 28. https://doi.org/10.3390/inorganics8040028 10.3390/inorganics8040028

[33] Ling D. Hyeon T.. Chemical design of biocompatible iron oxide nanoparticles for medical applications. Small 9 (2013) 1450-1466. https://doi.org/10.1002/smll.201202111 10.1002/smll.201202111 23233377

[34] Vargas-Ortiz J.R. Gonzalez C. Esquivel K.. Magnetic Iron Nanoparticles: Synthesis, Surface Enhancements, and Biological Challenges. Processes 10 (2022) 2282. https://doi.org/10.3390/pr10112282 10.3390/pr10112282

[35] Affrald R.J. Banu S.P.N. Arjunan D. Selvamani K.A. Narayan S.. Synthesis and Characterisation of Alginate Functionalized Gold Nanoparticles for Melamine Detection. BioNanoScience 13 (2023) 145-152. https://doi.org/10.1007/s12668-022-01050-5 10.1007/s12668-022-01050-5

[36] Sharmiladevi P. Akhtar N. Haribabu V. Girigoswami K. Chattopadhyay S. Girigoswami A.. Excitation wavelength independent carbon-decorated ferrite nanodots for multimodal diagnosis and stimuli responsive therapy. ACS Applied Bio Materials 2 (2019) 1634-1642. https://doi.org/10.1021/acsabm.9b00039 10.1021/acsabm.9b00039 35026897

[37] Geraldes C.F.G.C.. Rational Design of Magnetic Nanoparticles as *T*_1_–*T*_2_ Dual-Mode MRI Contrast Agents. Molecules 29 (2024) 1352. https://doi.org/10.3390/molecules29061352 10.3390/molecules29061352 38542988 PMC10974227

[38] Yue H. Zhao D. Tegafaw T. Ahmad M.Y. Saidi A.K. Liu Y. Cha H. Yang B.W. Chae K.S. Nam S.-W. Chang Y. Lee G.H.. Core-Shell Fe_3_O_4_@C Nanoparticles as Highly Effective *T*_2_ Magnetic Resonance Imaging Contrast Agents: In Vitro and In Vivo Studies. Nanomaterials 14 (2024) 177. https://doi.org/10.3390/nano14020177 10.3390/nano14020177 38251140 PMC10819740

[39] Gowtham P. Girigoswami K. Pallavi P. Harini K. Gurubharath I. Girigoswami A.. Alginate-Derivative Encapsulated Carbon Coated Manganese-Ferrite Nanodots for Multimodal Medical Imaging. Pharmaceutics 14 (2022) 2550. https://doi.org/10.3390/pharmaceutics14122550 10.3390/pharmaceutics14122550 36559045 PMC9782169

[40] Sharmiladevi P. Haribabu V. Girigoswami K. Sulaiman Farook A. Girigoswami A.. Effect of Mesoporous Nano Water Reservoir on MR Relaxivity. Scientific reports 7 (2017) 11179. https://doi.org/10.1038/s41598-017-11710-2 10.1038/s41598-017-11710-2 28894269 PMC5593907

[41] Li L. Mak K. Leung C. Chan K. Chan W. Zhong W. Pong P.. Effect of synthesis conditions on the properties of citric-acid coated iron oxide nanoparticles. Microelectronic Engineering 110 (2013) 329-334. https://doi.org/10.1016/j.mee.2013.02.045 10.1016/j.mee.2013.02.045

[42] Jiang B. Tang Y. Qu Y. Wang J.-Q. Xie Y. Tian C. Zhou W. Fu H.. Thin carbon layer coated Ti3+-TiO2 nanocrystallites for visible-light driven photocatalysis. Nanoscale 7 (2015) 5035-5045. https://doi.org/10.1039/C5NR00032G 10.1039/C5NR00032G 25697803

[43] Liu X. He L. Han G. Sheng J. Yu Y. Yang W.. Design of rich defects carbon coated MnFe2O4/LaMnO3/LaFeO3 heterostructure nanocomposites for broadband electromagnetic wave absorption. Chemical Engineering Journal 476 (2023) 146199. https://doi.org/10.1016/j.cej.2023.146199 10.1016/j.cej.2023.146199

[44] Chen X. Zhou Y. Han H. Wang X. Zhou L. Yi Z. Fu Z. Wu X. Li G. Zeng L.. Optical and magnetic properties of small-size core–shell Fe3O4@C nanoparticles. Materials today chemistry 22 (2021) 100556. https://doi.org/10.1016/j.mtchem.2021.100556 10.1016/j.mtchem.2021.100556

[45] Caruntu D. Caruntu G. O'Connor C.J.. Magnetic properties of variable-sized Fe3O4 nanoparticles synthesized from non-aqueous homogeneous solutions of polyols. Journal of Physics D: Applied Physics 40 (2007) 5801. https://doi.org/10.1088/0022-3727/40/19/001 10.1088/0022-3727/40/19/001

[46] Chaudhary S. Umar A. Bhasin K. Singh S.. Applications of carbon dots in nanomedicine. Journal of Biomedical Nanotechnology 13 (2017) 591-637. https://doi.org/10.1166/jbn.2017.2390 10.1166/jbn.2017.2390

[47] Thirumalai A. Girigoswami K. Prabhu A.D. Durgadevi P. Kiran V. Girigoswami A.. 8-Anilino-1-naphthalenesulfonate-Conjugated Carbon-Coated Ferrite Nanodots for Fluoromagnetic Imaging, Smart Drug Delivery, and Biomolecular Sensing. Pharmaceutics 16 (2024) 1378. https://doi.org/10.3390/pharmaceutics16111378 10.3390/pharmaceutics16111378 39598502 PMC11597131

[48] Haribabu V. Girigoswami K. Sharmiladevi P. Girigoswami A.. Water–Nanomaterial Interaction to Escalate Twin-Mode Magnetic Resonance Imaging. ACS Biomaterials Science & Engineering 6 (2020) 4377-4389. https://doi.org/10.1021/acsbiomaterials.0c00409 10.1021/acsbiomaterials.0c00409 33455176

[49] Gowtham P. Harini K. Thirumalai A. Pallavi P. Girigoswami K. Girigoswami A.. Synthetic routes to theranostic applications of carbon-based quantum dots. ADMET and DMPK 11 (2023) 457–485. https://doi.org/10.5599/admet.1747 10.5599/admet.1747 37937240 PMC10626517

[50] Abou Elfadl A. Ibrahim A.M.M. El Sayed A.M. Saber S. Elnaggar S. Ibrahim I.M.. Influence of α-Fe2O3, CuO and GO 2D nano-fillers on the structure, physical properties and antifungal activity of Na-CMC–PAAm blend. Scientific reports 13 (2023) 12358. https://doi.org/10.1038/s41598-023-39056-y 10.1038/s41598-023-39056-y 37524718 PMC10390538

[51] Yan Y. Tang H. Wu F. Wang R. Pan M.. One-step self-assembly synthesis α-Fe2O3 with carbon-coated nanoparticles for stabilized and enhanced supercapacitors electrode. Energies 10 (2017) 1296. https://doi.org/10.3390/en10091296 10.3390/en10091296

[52] Qasim M. Iqbal A.M. Khan M.T. Ghanem M.A.. Nanostructural Modification of Fe2O3 Nanoparticles: Carbon Coating for Enhanced Magnetic Behavior. physica status solidi (RRL)–Rapid Research Letters (2025) 2400230. https://doi.org/10.1002/pssr.202400230 10.1002/pssr.202400230

[53] Moeni M. Edokali M. Rogers M. Cespedes O. Tliba L. Habib T. Menzel R. Hassanpour A.. Effect of reaction and post-treatment conditions on physico-chemical properties of magnetic iron oxide nano-particles. Particuology 91 (2024) 155-167. https://doi.org/10.1016/j.partic.2024.02.006 10.1016/j.partic.2024.02.006

[54] Ludmerczki R. Mura S. Carbonaro C.M. Mandity I.M. Carraro M. Senes N. Garroni S. Granozzi G. Calvillo L. Marras S.. Carbon dots from citric acid and its intermediates formed by thermal decomposition. Chemistry–A European Journal 25 (2019) 11963-11974. https://doi.org/10.1002/chem.201902497 10.1002/chem.201902497 31254368

[55] Kasprzyk W. Świergosz T. Bednarz S. Walas K. Bashmakova N.V. Bogdał D.. Luminescence phenomena of carbon dots derived from citric acid and urea – a molecular insight. Nanoscale 10 (2018) 13889-13894. https://doi.org/10.1039/C8NR03602K 10.1039/C8NR03602K 29999091

